# Polluted Air Exposure Compromises Corneal Immunity and Exacerbates Inflammation in Acute Herpes Simplex Keratitis

**DOI:** 10.3389/fimmu.2021.618597

**Published:** 2021-02-25

**Authors:** Victor G. Sendra, Julia Tau, Gustavo Zapata, Romina M. Lasagni Vitar, Eduardo Illian, Pablo Chiaradía, Alejandro Berra

**Affiliations:** ^1^ Laboratorio Traslacional de Inmunopatología y Oftalmología, Departamento de Patología, Facultad de Medicina, Universidad de Buenos Aires, Cuidad Autónoma de Buenos Aires, Argentina; ^2^ Departamento de Química Analítica y Fisicoquímica, Facultad de Farmacia y Bioquímica, CONICET—Instituto de Bioquímica y Medicina Molecular (IBIMOL), Universidad de Buenos Aires, Cuidad Autónoma de Buenos Aires, Argentina; ^3^ Neurovirosis, Departamento de Virología, Instituto Nacional de Enfermedades Infecciosas (INEI), Administración Nacional de Laboratorios e Institutos de Salud (ANLIS) Malbrán, Cuidad Autónoma de Buenos Aires, Argentina; ^4^ Departamento de Oftalmología, Hospital de Clínicas, Universidad de Buenos Aires, Cuidad Autónoma de Buenos Aires, Argentina

**Keywords:** air pollution, herpes simplex keratitis (HSK), immune response, draining lymph nodes (dLNs), corneal immune cells

## Abstract

Air pollution is a serious environmental issue worldwide in developing countries’ megacities, affecting the population’s health, including the ocular surface, by predisposing or exacerbating other ocular diseases. Herpes simplex keratitis (HSK) is caused by the herpes simplex virus type 1 (HSV-1). The primary or recurring infection in the ocular site causes progressive corneal scarring that may result in visual impairment. The present study was designed to study the immunopathological changes of acute HSK under urban polluted air, using the acute HSK model combined with an experimental urban polluted air exposure from Buenos Aires City. We evaluated the corneal clinical outcomes, viral DNA and pro-inflammatory cytokines by RT-PCR and ELISA assays, respectively. Then, we determined the innate and adaptive immune responses in both cornea and local lymph nodes after HSV-1 corneal by immunofluorescence staining and flow cytometry. Our results showed that mice exposed to polluted air develop a severe form of HSK with increased corneal opacity, neovascularization, HSV-1 DNA and production of TNF-α, IL-1β, IFN-γ, and CCL2. A high number of corneal resident immune cells, including activated dendritic cells, was observed in mice exposed to polluted air; with a further significant influx of bone marrow-derived cells including GR1+ cells (neutrophils and inflammatory monocytes), CD11c+ cells (dendritic cells), and CD3+ (T cells) during acute corneal HSK. Moreover, mice exposed to polluted air showed a predominant Th1 type T cell response over Tregs in local lymph nodes during acute HSK with decreased corneal Tregs. These findings provide strong evidence that urban polluted air might trigger a local imbalance of innate and adaptive immune responses that exacerbate HSK severity. Taking this study into account, urban air pollution should be considered a key factor in developing ocular inflammatory diseases.

## Introduction

The ocular surface mucosa comprises the cornea, conjunctiva and tear film, and acts as the first barrier against external threats, including polluted components present in the air. Immunologically, the ocular surface is among the few tissues with immune privilege, deprived of immune reaction to the constant exposure of foreign antigens, allergens, and pathogens, keeping immunological homeostasis and corneal transparency (1, 2). Characterized by particulate matter (PM), urban air pollution is well known for causing adverse health effects. In the ocular surface, polluted air can affect vision by causing discomfort, redness, irritation, tear film deficiency and blurry vision (3, 4); however, the impact throughout the inflammatory eye diseases is unexplored. PM is composed of a heterogeneous mix of tiny solid or liquid particles suspended in the air and is identified as primarily responsible for the health outcomes associated with urban air pollution. PM can be denoted according to size as PM_10_, when diameter less than 10 μm, or PM_2.5_, when it is 2.5 (5). Traffic emissions make an important contribution to the PM2.5 composition in Buenos Aires and other big cities’ polluted air, causing adverse biological responses *in vivo* and *in vitro* (6–8). Our group and others have demonstrated *in vitro* that corneal and conjunctival epithelial cells increase the secretion of pro-inflammatory cytokines with redox imbalance under incubation with diesel exhaust particles (DEP) (the main component of urban PM); moreover, *in vivo* exposure to air pollution from Buenos Aires altered the ocular surface cellularity, redox imbalance and the inflammatory cytokines on the cornea (9–11). On the other hand, three studies have shown that air pollution increases the susceptibility of respiratory mucosa to the H1N1 influenza (12, 13) virus and respiratory syncytial virus (RSV) (14). Based on these previous findings, we will explore the potential effects of chronic air pollution exposure on cornea immunity in the common infectious disease, herpes simplex keratitis (HSK).

Simplex Virus type 1 (HSV-1), and it is the leading cause of blindness in developed countries (15). Like other herpes viruses, there are primary and recurrent forms of the disease. Typically, acute primary herpetic keratitis begins with the replication of HSV-1 in the corneal epithelium. An effective immune response is critical for controlling viral infections, but it can also cause corneal lesions, orchestrated mainly by neutrophils, dendritic cells, and CD4^+^ T cells (16–19). The mouse HSV-1 corneal infection model has been used extensively as an experimental HSK model to study immunopathological mechanisms in many conditions, including the environmental (20–23).

Health risks from long-term exposure to PM are much than those from short-term exposure, and represent more than the cumulative impacts of repeated short-term exposures (24–26). Toxicological studies support the epidemiological evidence showing several of possible biological mechanisms for the observed outcomes, such as systemic inflammation and vascular dysfunction (27). Based on this data, we hypothesized that long-term exposure to urban polluted air would induce an immunological imbalance of the ocular surface that could lead to decreased HSV-1 clearance, increasing the inflammatory response and worsening clinical severity.

To evaluate our hypothesis, we performed corneal HSV-1 infection on BALB/c mice exposed since birth to urban polluted air of Buenos Aires city, Argentina, to determine how urban air pollution exposure affects innate and adaptive immune responses during acute HSK.

## Materials and Methods

### Animals

Six to eight weeks old BALB/c mice were purchased from the Faculty of Veterinary of La Plata, Buenos Aires, Argentina. The protocol was approved by the Institutional Committee for the Care and Use of Laboratory Animals (CICUAL) from the University of Buenos Aires, in concordance to the Animal Care Guidelines from the National Institute of Health (USA) and the Association for Research in Vision and Ophthalmology (ARVO, USA) resolution on the use of animals in research.

### Polluted Air Chronic Exposure Mouse Model

Two air forced ventilated chamber systems located in a highly populated area of Buenos Aires City (latitude 34°35’51’’S and longitude 58°23’56’O) were used as previously described (11). One chamber received urban airflow from Buenos Aires city and the other one received filtered indoor airflow. The exposure protocol comprised exposures for 8 h/day during 2018/19. Briefly, the non-exposed group received an identical protocol with a high-efficiency particulate air filter (Mark 80 pre-filter followed by a C-cell compact filter, Microfilter, Argentina) positioned in the inlet valve to remove particulate matter (PM) from the filtered air stream. Mice were housed in a 12-h light/12-h dark cycle and were fed food and water ad libitum under controlled temperature and humidity conditions. During 2018, the experimental year, the mean level of particulate matter (PM10) in this area was lower than 54 μg/m^3^ according to the Environmental Protection Agency of Buenos Aires City (28), while PM2.5 were over 10 µg/m^3^ in daily average according to our air quality measurement (AirVisual pro, IQAir, CA, USA).

### Herpes Simplex Keratitis Mouse Model

On 7-week-old male BALB/c mice corneal infection was performed under deep anesthesia induced by intraperitoneal administration of a combination of ketamine (50 mg/kg, Parke-Davis, Morris Plains, NJ) and xylazine (10 mg/kg, Mobay, Shanee, KA). The right cornea of all mice was scarified by making 5 x 5 lines with a 30-gauge needle under dissection microscope (Carl Zeiss, Germany). Subsequently, 5-µl drop of 2 x 10^7^ PFU/ml HSV-1 KOS strain stock solution was placed on the eye (1 x 10^5^ PFU/eye), making a gentle massage on the eyelids.

Four groups were evaluated: 1) Experimental Normal Control: mice (n=24) scarified and exposed clean air, 2) Experimental Normal Polluted Control: mice (n=24) scarified and exposed to polluted air of Buenos Aires city since they were born, 3) Clean Air HSK: mice (n=24) infected and exposed to clean filtered air, 4) Polluted Air HSK: mice (n=24) infected and exposed to polluted air of Buenos Aires city since they were born.

The mice were maintained in the racks until 11 days post infection (dpi). All mice were clinically evaluated at 0, 5, 7, and 11 dpi. Eye swab material was collected to determine cytokines and viral DNA at 1, 2, 3, 5, 7, and 11 dpi. The swabs samples were taken in 10 µl of saline solution per cornea. Normal uninfected and 3 dpi corneas were excised and process for immunofluorescence assay. Similarly, normal uninfected and HSV-1 infected at days 7 and 11 corneas and dLNs were collected for flow cytometric analysis. Lymph node area was measured by using ImageJ software (NIH, Bethesda, MD).

### Clinical Scoring

The severity of HSK was determined and photographed in a blinded fashion under a binocular microscope at days 0, 5, 7, and 11 dpi. A scoring system based on corneal opacity and neovascularization was used as follows: Corneal opacity: 0 = clear cornea, 1 = slight corneal turbidity, some iris detail visible, 2 = moderate corneal opacity with iris detail obscured, 3 = severe corneal opacity and 4 = severe corneal opacity impeding the observation of the iris, with signs of corneal necrosis or perforation; Neovascularization: 0 = no vessels visible, 1 = vessel enlargement with corneal invasion, 2 = 10%–30% of the cornea involved, 3 = 30%–60% of the cornea involved, with vessels present in the center of the cornea and 4 = 60%–100% of the cornea involved, invaded by a great number of vessels. The BALB/c mice that showed opacity score ≥ 2 and/or neovascularization score ≥ 1 were considered with HSK.

### Determination of Viral DNA

Real Time PCR was performed to determinate the amount of viral DNA in the cornea swabs. First, the DNA of the samples was extracted using a spin-column based QIAamp Mini Kit (Qiagen, Hilden, Germany) following the manufacturer extraction protocol. Each sample consist of a pool of cornea washes (n=3, three pools per group were measured). The Real-Time PCR was performed with oligonucleotide primer pairs and probes specific for the type common region of HSV-1 and HSV-2 glycoprotein B (gB), as reported previously (29). The primers used were HSV-FP (5´- TCC CGG TAC GAA GAC CAG-3´) and HSV-RP (5´- AGC AGG CCG CTG TCC TTG-3´), and the probe was HSV-TCP (5´-FAM-TGG TCC TCC AGC ATG GTG ATG TTG/C AGG TCG-TAMRA-3´). The protocol used was reported previously to determine Ct values and compare between samples (30). Amplification was carried out in an Applied Biosystem Sequence Detector 7500, programmed for a four-step protocol: 2 min of incubation at 50°C for AmpErase activation, 10 min at 95°C for polymerase activation and for 45 cycles: 15 s at 94°C for denaturation, 60 s at 58°C for annealing, extension and data collection. Each 50 µl of PCR reaction mix contained: 10 µl of purified DNA, 833 nM concentrations of each primer and 100 nM probe in 1x TaqMan universal PCR master mix (Applied Biosystems, Branchburg, New Jersey USA). Negative controls were included in the extraction process every 20 clinical samples. All negative samples were tested twice.

### Cytokine Quantification

The pools of the corneal washes obtained for each group (n=3, three pools per group were measured) were used to determine the secretion of TNF-α, IL-1β, IL-6, IFN-γ, and CCL2 using ELISA kits (BD Biosciences, New Jersey, USA and BioLegend, San Diego CA, USA) following the manufacturer’s technical specifications.

### Corneal Immunofluorescence Assay and Confocal Imaging

Normal and HSV-1 infected corneas at 3dpi were excised, cleaned and fixed with 4% paraformaldehyde. After washing, flat-mounted fixed corneas were washed with PBS 3 times and then blocked in 2% bovine serum albumin (BSA) followed by incubation with 1% anti-CD16/CD32 Fc receptor (FcR) mAb (BioLegend, San Diego, CA, USA) for 30 min at RT. Next, samples were stained with fluorophore-conjugated antibodies for bone marrow-derived cells, dendritic cells, macrophages and the activation cell marker by using anti-CD45 (FITC) (clone 30-F11) (BioLegend, San Diego, CA, USA), anti-CD11c (PE) (clone NL3) (BD Pharmingen, San Jose, CA), anti-F480 (AF647) (clone BM8) (BioLegend, San Diego, CA, USA) and anti-MHC class II (AF647) (clone M5/114.15.1) (BioLegend, San Diego, CA, USA) overnight at 4˚C. After three washes with PBS, corneas were mounted and imaged by using FV-1000 confocal microscope (Olympus Co, Tokyo, Japan) provided by Sistema Nacional, Ministerio de Ciencia, Tecnología e Innovación, Argentina. For quantification purposes, three images from the periphery and central flat-mounted corneas per animal were taken. Quantification of cells was performed using ImageJ software (NIH, Bethesda, MD).

### Flow Cytometric Analysis

Briefly, pools of 2–3 corneas were collected at 7 and 11 dpi HSK and stored in PBS-EDTA at 37°C for 15 min at 4°C. Within hours, corneas were triturated in small pieces followed by a digestion in 84 U collagenase type 1 (Sigma-Aldrich Corp., St. Louis, MO. USA) for 2 h at 37°C. Afterward, a single-cell suspension were filtered through a 40-μm cell strainer cap (BD Labware, Bedford, MA, USA) and washed. The cell suspensions were then stained for surface markers for innate immune cell populations by using fluorophore-conjugated antibodies anti-CD45 (clone 30-F11), anti-CD3 (clone 14A2), anti-Gr-1 (clone RB6-8C5), anti-F4/80 (clone BM8) (BioLegend, San Diego, CA, USA) and anti-CD11c (clone HL3) (BD Pharmingen, San Jose, CA). To determine regulatory T cells (Tregs), corneal cells were stained with a combination of fluorophore-conjugated antibodies against CD3, CD4, and CD25 cell surface markers followed by fixation and permeabilization for intracellular staining by using Fix/Perm buffer set (Thermo Scientific). Then, cells were stained for FoxP3 marker with fluorophore-conjugated antibody (BioLegend, San Diego, CA, USA).

T cell response analysis in local submandibular draining lymph nodes (dLN) were performed by excising dLNs at 7 dpi and meshing to obtain single cells suspension. T helper (Th) -1 and -17 cells were stimulated in presence of phorbol 12-myristate 13-acetate (PMA, 50 ng/ml; Sigma-Aldrich) and Ionomycin (1 µg/ml; Sigma-Aldrich) for 5 h in the presence of a protein transport inhibitor, Brefeldin A (GolgiPlug, BD Biosciences, San Jose, CA). Then, cells were stained for CD3 and CD4 cell surface marker followed by intracellular staining for the cytokines IFN-γ or IL-17A (Th1 and Th17 response respectively) by fixing with 4% paraformaldehyde and permeabilizing with 0.1% saponin/PBS. For Tregs analysis, cell suspensions were stained with fluorophore-conjugated antibody for CD3, CD4 and CD25 cell surface marker (BioLegend, San Diego, CA, USA) followed by intracellular staining, using the intracellular fixation & permeabilization buffer set (ThermoFisher Scientific Grand Island, NY, USA). Then, cells were stained for the transcription factor, FoxP3 with fluorophore-conjugated antibody (BioLegend, San Diego, CA, USA). Cells population were determined on a flow cytometer (FACSAria II; BD Biosciences, San Jose, CA) provided by Sistema Nacional, Ministerio de Ciencia, Tecnología e Innovación, Argentina. The data were analyzed by using FlowJo software (TreeStar, Ashland, OR, USA). The strategy for analysis was to initially gate on population cells and then the CD45^+^ cells. These cells were further evaluated for T cell marker CD3^+^, or for macrophage marker F4/80^+^, or neutrophil marker GR-1^+^, or dendritic cell marker CD11c^+^. For T cell response, total CD45+ cell was gated for CD3 and CD4 cell surface markers, and then Th1 and Th17 response were identified as IFN-γ+ or IL-17A+ respectively, while Tregs were identified based on the expression of CD25 cell surface marker and the transcription factor, FoxP3.

### Statistical Analysis

The values obtained from clinical scoring were analyzed by Kruskal–Wallis test and Mann Whitney test (α=0.05) as a post-hoc test. The values obtained from Real Time-PCR, ELISA, immunofluorescence assay and flow cytometry were analyzed by One-Way ANOVA test (p=0.05) and Bonferroni test (p=0.05) as a post-hoc test. All statistical analyses and figures were performed with the SPSS 17.0 (Sun Microsystems, Inc., CA, USA) and Prism (GraphPad software, San Diego, CA, USA).

## Results

### Polluted Air Exposure Increases Corneal Opacity, Neovascularization, and Viral Load After Acute Herpes Simplex Virus Type 1 Corneal Infection

To study the effect of chronic exposure to air pollution during acute HSK, we used an *in vivo* mouse model, housing them in a chamber exposed to either polluted air from Buenos Aires or filtered clear air in combination with a well-described mouse model for acute HSV-1 keratitis.

**Figure 1:**
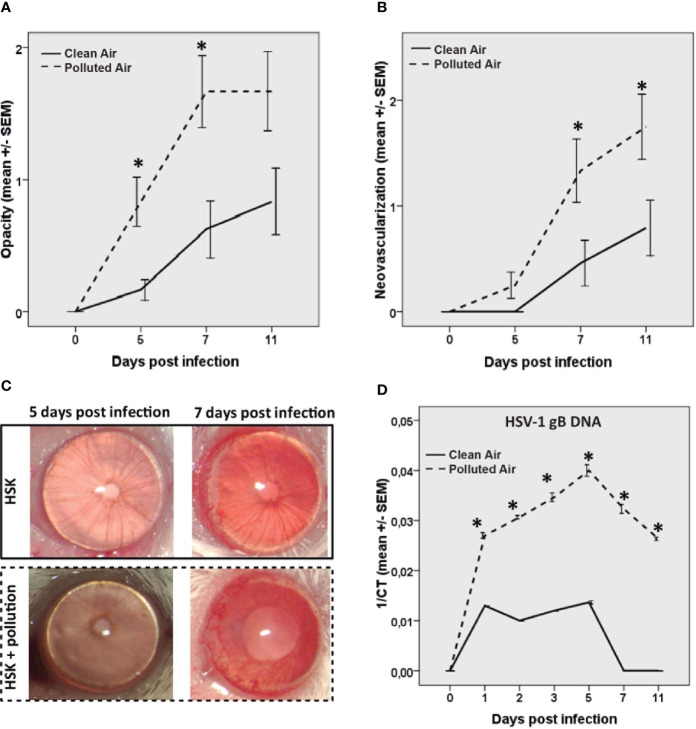
Corneal opacity **(A)** and corneal neovascularization **(B)** were measured and compared between the groups during acute herpes simplex keratitis (HSK) until day 11. Representative corneas pictures of HSK mice at 5 and 7 dpi are shown **(C)**. HSV-1 gB DNA measured by real time PCR at different time points of the acute phase of the infection **(D)** in mice exposed to polluted (dashed line —) and clear air (continued line —). *p < 0.05, differences between groups are statistically significant.

Given that HSV-1 infection triggers inflammation and neovascularization affects corneal transparency during HSK development, we quantified the animals’ HSK severity regarding corneal opacity and neovascularization. Our results showed that HSK mice exposed to polluted air had a significant increase in clinical scores, opacity, and neovascularization compared to the group exposed to clear filtered air (**Figures 1A–C**). Mouse corneal opacity significantly increased in polluted air-exposed group at 5 dpi (p=0.004) and 7 dpi (p=0.008) while neovascularization significantly increased at 7 dpi (p=0.04) and 11 dpi (p=0.04) compared to the group exposed to clean air. There were no clinical signs of disease (opacity or neovascularization) in the scarified uninfected control group for experimental conditions observed on any day.

Next, we evaluated the amount of HSV-1 DNA in mice cornea at different times post-infection (**Figure 1D**). The HSV-1 DNA increased in both infected mice groups with the same frequency, from one dpi to five dpi, and decreased from 5 dpi to 11 dpi (**Figure 1D**). The corneal viral DNA detected here is consistent with previous publications, where the replicating virus was detectable in the cornea during HSV-1 infection until days 5 and 6 (16). Next, we observed that mice exposed to polluted air showed a significantly higher amount of HSV-1 DNA compared to clear air-exposed mice (p<0.01) until the end of the experiment at 11 dpi. HSV-1 DNA was not detectable in the experimental control group on any day. This data demonstrates that air pollution has a deleterious effect during HSK by worsening clinical severity and increasing the viral load.

Additionally, we observed that none of the mice exposed to clear air developed clinical signs of HSK until day 5; however, at day 7 and 11 post-infection (dpi) with HSV-1, 17% (4/24) and 33% (8/24) of the mice, respectively, developed clinical signs of disease. Interestingly, 17% (4/24) of mice exposed to polluted air showed clinical signs as early as 5 dpi, followed by 54% (13/24) at 7 dpi, and 58% (14/24) at 11 dpi of HSK (**Supplementary Figure 1A**). This evidence clearly indicates that chronic exposure to air pollution increases acute HSK incidences based on clinical severity.

#### Polluted Air Exposure Alters Pro-Inflammatory Cytokines Release After Acute Herpes Simplex Virus Type 1 Corneal Infection

**Figure 2:**
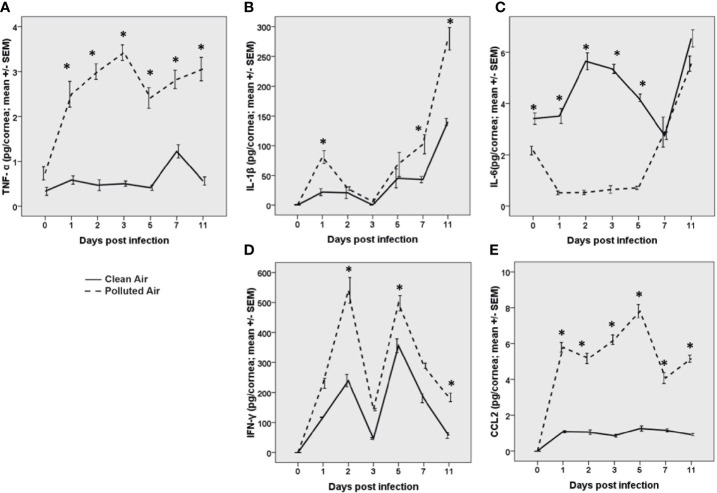
Pro-inflammatory cytokine quantification of TNF-α **(A)**, IL-1β **(B)**, IL-6 **(C)**, IFN-γ **(D)**, and the chemokine CCL2 **(E)** measured by ELISA during acute herpes simplex keratitis (HSK) in mice exposed to polluted (dashed line —) and clear air (continued line —)**** at days 0, 1, 2, 3, 5, 7, and 11 post-infection. *p < 0.05, differences between groups are statistically significant.

Corneal HSV-1 infection trigger of the innate immune response by activation of antigen presenting cell (APCs), production of pro-inflammatory cytokines and chemokines early after infection. Due to the differences found in innate immune cells between the infected mice, we further analyzed the release of the main cytokines known to be upregulated after HSV-1 corneal infection: TNF-α, IL-1β, IL-6, IL-10, IFN-γ, and CCL2. We observed and increased production of TNF-α, IL-1β, IFN-γ, and CCL2 (p<0.05) in air polluted exposed mice after HSV-1 infection compared with clean air exposed group (**Figures 2A, B, D, E**, respectively). TNF-α functions in the mediation of the acute-phase response, chemotaxis, and activation of inflammatory and antigen-presenting cells (APCs) (31, 32). The secretion of IL-1β and IL-6 has been linked to the recruitment of PMN to the virus infection site (33, 34), while increased IFN-γ production is indication of T cell response and is involved in the initial monocyte recruitment as well (35). The chemokine CCL2 are associated with the influx of activated T cells, PMN and monocytes into the infected cornea (35, 36). Interestingly, IL-6 was significantly increased only in mice exposed to filtered air. However, IL-6 levels were similar in both groups since day 7 dpi (**Figure 2C**). No IL-10 release was detected in both groups at any time. Together, these results demonstrated that polluted air exposure promote pro-inflammatory cytokines production on the site of infection, contributing to intensify the inflammatory response.

### Polluted Air Exposure Increases Corneal Immune Cells Number in Normal Uninfected and Early Acute Herpes Simplex Virus Type 1 Keratitis

**Figure 3:**
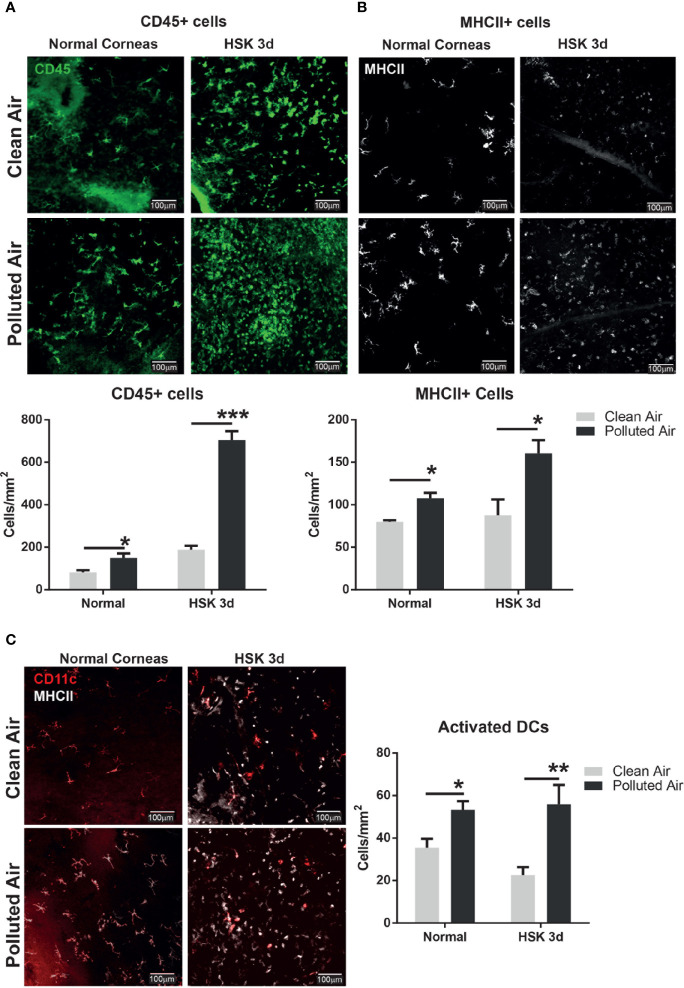
Corneal immune cell infiltration analysis by immunofluorescence in normal uninfected and early HSV-1 infected corneas. Whole mount corneas staining and quantification for leucocytes marker CD45 **(A)** and activation marker MHCII **(B)** in normal uninfected corneas and during early HSK at day 3 from mice exposed to clean air (top panel, gray bars) or polluted air (bottom panel, black bars). Whole mount corneas staining and quantification for dendritic cells marker CD11c and activation marker, MHCII **(C)** in normal non-infected and early HSK at day 3 from mice exposed to clean air (top panel, gray bars) or polluted air (bottom panel, black bars). Graph results are presented as mean ± standard error of the mean (SEM). *p < 0.05, **p < 0.01, ***p < 0.001, differences between groups are statistically significant.

Given the importance of the corneal immuneprivilege maintenance in the inflammatory infections, we next explored the corneal immune cells in normal and early day 3 of HSV-1 infection when no corneal inflammation is clinically evident in all groups (**Figure 1A**), to determine if the chronic exposure to polluted air have implications in resident immune cells balance that can explain the increased susceptibility to HSK observed at later days. Corneas from normal non-infected and after 3d HSV-1 infection were excised, flat-mounted and stained by immunofluorescence (IF) for CD45 (bone marrow derived cells) and MHCII (activation) to determine the corneal immune cells in normal and early stage of acute HSK at day 3 (**Figures 3A, B**, respectively). Normal non-infected flat-mounted corneas showed increased of immune cells (CD45+) (150 vs. 82 cells/mm^2^, p=0.036) in activation state (MCHII+) (108 vs. 80 cell/mm^2^, p=0.0012) in mice exposed to polluted air and compared to clean air. Similarly, we observed a significant higher influx of immune cells (704 vs. 190 cells/mm^2, p<0.001) in activation state (160 vs. 88 cells/mm^2, p=0.023) during early HSK at 3dpi in mice exposed to polluted air compared to mice exposed to clean air. More interestingly, we observed and increased on activated dendritic cells (CD11c+) in normal corneas (53 vs. 35 cell/mm^2, p=0.01) and early HSK at 3 dpi (56 vs. 22 cells/mm^2, p<0.01) (**Figure 3C**) after exposed to polluted air compared to clean air respectively; however, no significant differences were observed in macrophages populations (**Supplementary Figure 1B**). In addition, flow cytometry assay was performed in pool of normal corneas with comparable results, showing an increase of CD45 cells (19.4% vs. 13%) and activated dendritic cells (CD11+, MHCII+) (30% vs. 21%, respectively) in corneas exposed to polluted air compared to those exposed to clean air (**Supplementary Figures 1Cii–iii**). Altogether these results show that chronic exposure to air pollution induce an imbalance in corneal resident immune cells characterized by an increased number of activated DCs, turning to an immunogenic condition that is potentially affecting the immune homeostasis.

### Polluted Air Exposure Increases Immune Cells Influx During Acute Herpes Simplex Virus Type 1 Corneal Infection

Innate immune cells such as inflammatory monocyte and dendritic cell have been shown to reduce the viral load in HSV-1 infected corneas (35, 37). More importantly, the damage to corneal tissue is caused largely because of the overwhelming number of neutrophils in the inflamed cornea (38).

**Figure 4:**
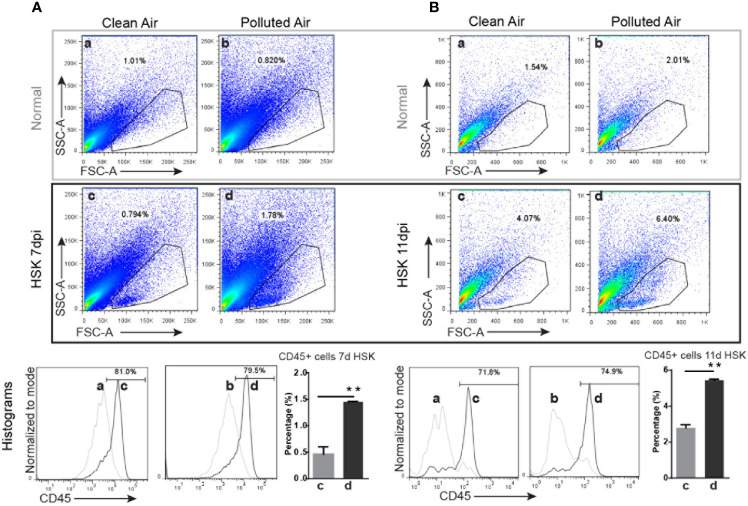
Corneal immune cell infiltration analysis by flow cytometry in normal uninfected and in HSV-1 infected corneas exposed to clean or polluted air. A gate was selected based on size, morphology and granularity (SSC vs. FCS) for normal uninfected and HSV-infected corneas at days 7 **(A)** and 11 **(B)** (top gray and bottom black panels respectively) from mice exposed to clean and polluted air (left and right respectively columns) were analyzed for cell infiltration. Then, the percentage of leucocyte (CD45 positive cells) for each gate; normal uninfected mice exposed to clean or polluted air (a and b respectively) and corneal HSV-1 infected mice exposed to clean or polluted air (c and d respectively). Bar graph shows corneal CD45+ cells quantification during HSK at days 7 and 11 from mice group exposed to clean (a) or polluted air (d). Graph results are presented as mean ± standard error of the mean (SEM). *p < 0.05, differences between groups are statistically significant.

Based on the differences in the clinical outcomes, the amount of HSV-1 DNA and the number of corneal resident immune cells observed in mice exposed to polluted air compared to clean air, we further characterized the inflammatory immune cells in the cornea during the clinical stage of primary HSK. We analyzed corneal immune cells by flow cytometry from HSV-1 infected mice at days 7 and 11 and compared between mice chronically exposed to clean filtered and polluted air. The immune cells were characterized for size, morphology, granularity and CD45 positive (pan-leukocyte marker). Overall, we observed a high infiltration of cells in HSV-1 infected corneas (**Figures 3B, D**) compared to normal non-infected corneas (**Figures 4A, C**). These results were not surprising, but we observed a significantly increased between mice exposed to polluted air compared to non-exposed infected group at 7 and 11 dpi (d vs. b, **Figures 4A, B**, respectively) (**Supplementary Figures 4A, B**). Thus, polluted air exposed mice showed significantly high amount of CD45+ cells compared to clean air exposed during HSK at day 7 (1.45 vs. 0.46% respectively, p<0.05) (**Figure 4A**) and day 11 (5.45% vs. 2.79%) (**Figure 4B**). Both, experimental control groups showed similar basal levels of immune CD45 positive cells in the histograms (a and b, gray lines) compared to 80% at day 7 and 72% at day 11 in HSK groups (c and d, black line).

**Figure 5:**
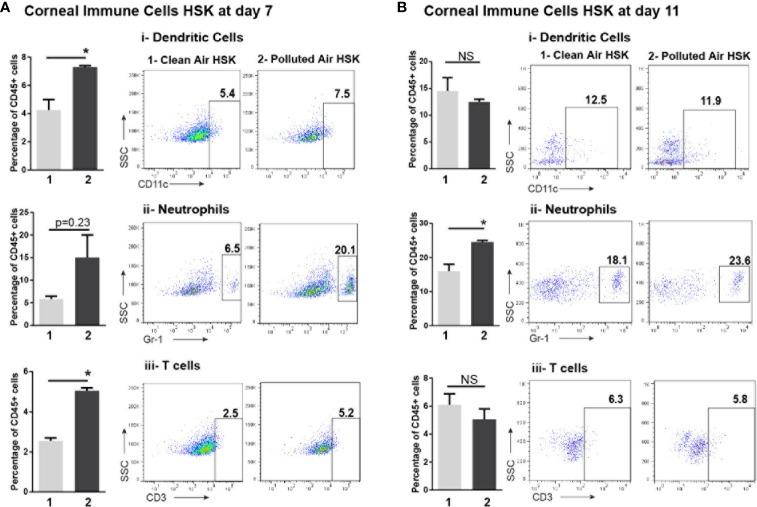
Corneal immune cell subpopulations analysis by flow cytometry during acute HSV-1 keratitis mice exposed to clean or polluted air. The gate for total leucocytes CD45+ were characterized for dendritic cell as CD11c+, neutrophils and inflammatory monocytes as GR-1+ and T-cell CD3+ markers in mice at days 7 **(A)** and 11 **(B)** during acute HSV-1 keratitis exposed to clean (1) or polluted air (2). Graph results are presented as mean ± standard error of the mean (SEM). *p < 0.05, differences between groups are statistically significant. NS: differences between groups are not statistically significant.

Next, to study more in details the corneal immune cells subpopulation involved in acute HSK after exposure to air pollution, the total immune cells CD45+ were gated and phenotyped for CD3+ (T cell marker), F4/80+ (macrophage marker), Gr-1+ (neutrophils and inflammatory monocytes marker), CD11c+ (dendritic cell marker), and NK1.1+ (NK cell marker). At 7 dpi, Gr-1, CD11c and CD3 markers were higher in HSK mice exposed to polluted air compared to mice exposed to clean air: Gr-1+ cells (15% vs. 6%, P = 0.23), CD11c+ dendritic cells (7.3% vs. 4.2%, P< 0.05) and CD3+ T cells (5.0% vs. 2.5%, P<0.05) (**Figure 5A**). At 11 dpi HSK mice exposed to polluted air showed higher amount of Gr-1+ cells (24% vs. 16%, P<0.05) but no significant differences in dendritic cells or T cells compared to HSK mice exposed to clean filtered air (**Figure 5B**). No differences on macrophages or NK cells populations was observed at any time during HSK between mice groups exposed to clean or polluted air.

### Polluted Air Exposure Promote Effector T Cell Response in Draining Lymph Nodes During Acute Herpes Simplex Virus Type 1 Corneal Infection

Adaptive immunity and specially the balance of T cell effector [T helper (Th) -1 and -17] with regulatory T cells (Tregs) play a critical role in the development of corneal lesions and its duration by either promoting a pro-inflammatory or a tolerogenic response respectively. Moreover, the presence of Tregs in corneal infection is associated with less inflammation (39). Thus, we next explored the changes in the adaptive immunity in local draining lymph nodes (dLNs) to determine the type of T cell response involved during acute HSK on mice exposed to polluted air.

**Figure 6:**
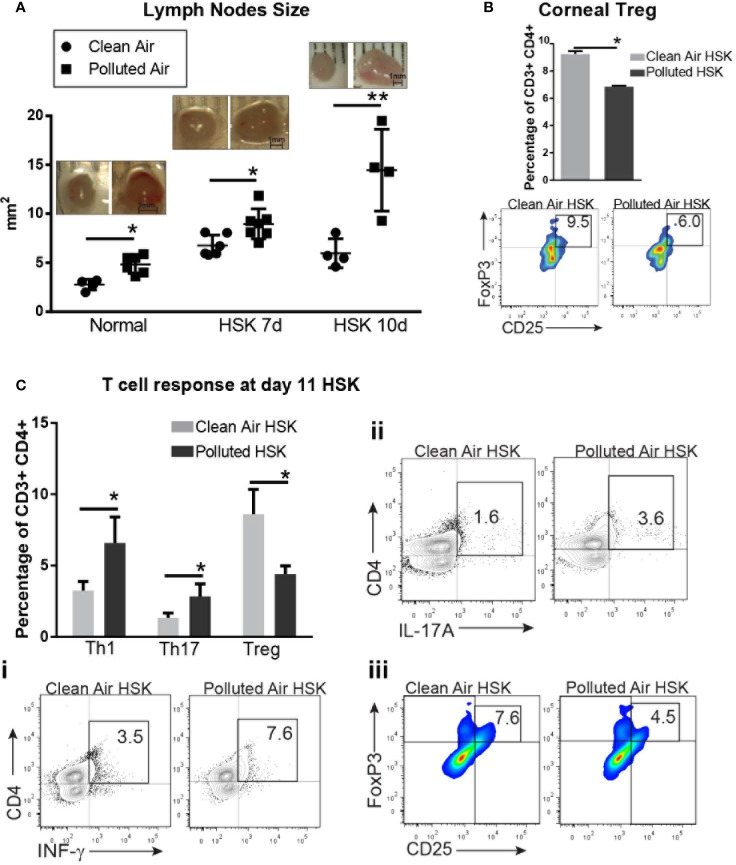
Corneal draining lymph node immune cell subpopulations analysis by flow cytometry during acute HSV-1 keratitis mice exposed to clean or polluted air. Draining lymph nodes size are expressed in mm2 in normal mice and during HSV-1 keratitis from mice exposed to clean (●) or polluted (◾) air **(A)**. Corneal regulatory T cells, Tregs (FoxP3+, CD25+) from HSV-1 infected corneas at 10dpi from mice exposed to clean (gray bar) or polluted air (black bar) expressed as percentage of gated CD3+ CD4+ T cells **(B)**. T cell response Th1 (IFN-γ+) (i), Th17 (IL-17A+) (ii) and regulatory T cells, Treg (CD25+, FoxP3+) (iii) in local lymph nodes from HSV-1 infected corneas at 10 dpi from mice exposed to clean, n=4 (gray bar) or polluted air, n=5 (black bar) expressed as percentage of gated CD3+ CD4+ T cells **(C)**. Graph results are presented as mean ± standard error of the mean (SEM). *p < 0.05, **p < 0.01, differences between groups are statistically significant.

First, we observed a significant enlargement of dLN from mice exposed to air pollution compared to exposed to clean air in all the conditions: normal non-infected mice (4.8 mm2 vs. 2.8 mm2, p<0.01 respectively), and during HSK at day 7 (8.9 vs. 6.7, p=0.013) and 11 (14.4 vs. 6.0, p<0.01) (**Figure 6A**).

Next, we characterized the CD4+ T cell response to determine whether effector T cells or regulatory T cells are promoted during HSK in polluted air-exposed mice. At day 11 post infection, we observed a significantly increased T helper response (Th) Th1 (6.5% vs. 3.2%, p<0.01) (**Figure 6Ci**) and Th17 (2.8% vs. 1.3%, p<0.01) (**Figure 6Cii**) with decreased regulatory T cells response (Treg) (4.4% vs. 8.6%, p<0.01) (**Figure 6Ciii**) in polluted air mice group compared to clean air group. Although we previously found no changes in T cells in HSV-1 infected corneas (**Figure 5Biii**), we did notice that polluted air exposed corneas had lower Tregs compared to clean air group (9.2% vs. 6.8%, p<0.01) (**Figure 6B**), in concordance with the low Treg production in dLNs. In order to compared the type of T cell response during chronic exposure without corneal infection and in another time point of HSK we performed the same determination for in T cell response by flow cytometry. Our data showed that in dLNs from mice exposed to air pollution had increased Th1 response (11.2% vs. 3.3%, p<0.01) but no significant changes in Th17 or Treg population compared to mice exposed to lean air (**Supplementary Figure 2**). At day 7 dpi, we observed a significantly increased Th1 response (11.2% vs. 3.3%, p<0.01) (**Supplementary Figure 2i**) with decreased regulatory T cells response (Treg) (5.6% vs. 7.0%, p<0.01) (**Supplementary Figure 2iii**) in polluted air mice group compared to clean air exposed group.

Collectively, our data demonstrated that chronic exposure to air pollution provoke an increased clinical keratitis severity, viral load and corneal inflammation; promoting immune cell recruitment into the cornea, mainly dendritic cells, in early days and the prevalence of T cells, neutrophils and inflammatory monocytes later days. Also, polluted air exposure promotes effector T cell response Th1 and Th17 over regulatory T cells in dLNs during acute HSK.

## Discussion

Fifty-five percent of the world’s population currently resides in urban areas and is projected to reach 68% by 2050. Because of the environmental impact and the threat to the population’s health, air pollution is an increasing issue for big urban cities. The data concentration levels of particular matter <2.5 µm (PM2.5) reported in Buenos Aires are determined by traffic emissions and are the main component of air pollution (7). People living in urban areas with high exposure to PM frequently experienced ocular discomfort, including a burning sensation (4), tear film abnormalities, reduced tear break-up time and subclinical changes in the ocular surface, including goblet cell hyperplasia in the tarsal conjunctiva (40, 41). Further, there is a direct association between air pollution and the course of several diseases, including allergies, asthma, autoimmune diseases, etcetera (42–44).

Like other mucosal tissues, the cornea is permanently exposed to the environment and can serve as the entry portal for potential pathogens. Thus, it is highly likely that chronic exposure to air pollution may have a deleterious impact on the course of ocular infectious diseases.

In developed countries, HSK is the predominant cause of human blindness after cataracts, mainly due to its recurrent nature (37).

The pathogenesis of herpetic keratitis is complex and is still not fully understood. According to the current knowledge, corneal scarring and vascularization result from chronic inflammatory reactions against HSV antigens; thus, it is important to understand the implications of urban air pollution exposure from megacities in the anti-HSK immune responses. Here, we studied the impact of chronic exposure to air pollution during acute HSK by using a mouse model of herpes simplex keratitis (HSK) combined with chronic air pollution exposure. Given that in humans, primary HSV-1 infections are difficult to study because they are usually asymptomatic, the mouse models of HSK are an excellent experimental model to study herpes simplex keratitis pathogenesis in the acute phase (45). Additionally, our mouse model for studying air pollution exposure is a valid approach for studying the *in vivo* effects or the chronic, whole-body exposure to inhalation of urban polluted air (8). Thus, by combining both mouse models, we were able to study the *in vivo* effects of polluted air during acute HSK.

We evaluated the corneal clinical outcomes, the amount of HSV-1, the presence of pro-inflammatory cytokines, and immune cell characterization in corneas and local draining lymph nodes during HSK from mice exposed to air pollution from Buenos Aires and compared them to mice exposed to clean air. Our results show that mice exposed to polluted Buenos Aires air since birth developed a more severe clinical HSK with higher corneal opacity and corneal neovascularization compared to non-exposed mice. It is known that a larger amount of HSV-1 in the first 5 days post-infection is correlated with increased HSK severity between 5 and 11 dpi (23).

In this study, we observed that HSV-1 DNA was significantly higher in polluted air-exposed mice than clean air-exposed mice, leading to an increased inflammatory response with higher corneal opacity and neovascularization. This result shows a diminished HSV-1 clearance capacity in mice exposed to polluted air, suggesting an impairment on the ocular defense mechanisms.

As both effectors and regulators of ocular HSV-1 immunity, cytokines have an important role in the immune response (22). A number of cytokines were detected in the corneas of HSV-1-infected mice, including TNF-α, IL-1β, IL-6, IFN-γ, and CCL2 during acute infection or following latent virus reactivation (46). Our results indicate that chronic exposure to urban polluted air significantly increases HSK severity and increasing levels of TNF-α, IL-1β, IFN-γ, and CCL2, while levels of IL-6 decrease during the acute phase of HSK. Several chemokines such as CCL2 are promptly up-regulated in the cornea following HSV-1 replication in the epithelial layer (16). CCL2 from HSV-1-infected keratocytes attracts CD4^+^ T cells into the cornea (36), so the overproduction of CCL2 in animals exposed to the particulate matter could lead to an increasedCD4^+^ T cells, which releases more inflammatory molecules such as IFN-γ, which could explain the observed HSK severity. Moreover, the observed increased CD3^+^ T cells with decreased Tregs in polluted air-exposed corneas during HSK suggests a deficient regulatory function of Tregs that may exacerbate corneal inflammation.

Corneal epithelial cells may produce MMP-9 (a known angiogenesis factor) in response to inflammatory cytokines such as TNF-α (16). The increased corneal neovascularization observed here could be related to the increase of TNF-α secretion in line with previous findings during HSV-1 infection (47). In addition, increased viral load led to a major influx of immune cells, which led to an increase of TNF-α secretion by keratinocytes and immune cells (48).

IL-6 is highly expressed in the cornea during HSK (49) and it appears to be involved in controlling the virus’ replication during acute infection (31, 50), with immunomodulatory functions (31). Our data showed lower levels of IL-6 at the baseline and then significantly decreased at early HSK in mice exposed to polluted air, in concordance to the high amount of HSV-1 DNA and inflammation. Thus, this data support that IL-6 may have anti-viral and anti-inflammatory functions that decrease in mice exposed to polluted, air reducing the viral clearance and promoting corneal inflammation. This observation is in line with previous reports showing that DEP induced impairments in the immune cells functions by reducing IL-6 secretion (51).

On the other hand, our previous work demonstrated that our mouse model of air pollution exposure showed increased oxidative stress in corneal epithelial cells (11), potentially enhancing the susceptibility to viral infection and replication despite the increased antiviral mediator production like interferons (52).

To explore the corneal immune cells homeostasis after chronic exposure to air pollution, we determined resident corneal immune cell population changes by immunofluorescence assay. It is well known that corneal resident antigen-presenting cells (APCs) such as dendritic cells (DCs) are involved in the immuneprivilege maintenance, patrolling the cornea for any potential threat. In addition, DCs are considered the main professional antigen-presenting cells (APCs), playing a pivotal role in preventing corneal damage during HSK (17). Our findings showed an increased number of activated corneal immune cells, especially DCs in normal non-infected corneas exposed to air pollution that persist during the early stage of HSK, suggesting that this pre-condition correlates with an increased susceptibility for a severe HSK. It is worth noting that no changes in the clinical outcomes or pro-inflammatory cytokines were observed, regardless of the difference in activated corneal immune cells—including DCs—in non-infected mice exposed to air pollution. This data suggests that activated DCs may play a key role in the loss of the immune homoeostasis by increasing the potential immunogenic response during HSK. Further, we observed an abnormally increased number of leucocytes characterized by increased activated DCs during later HSK after day 5, which correlates with increased HSK severity (53). Another important point is the unchanged corneal macrophage population; while they are the key players in the immune response to pollutants in other tissues (51, 54), our data here indicates that corneal resident dendritic cells are the main APCs players in driving the immune response in corneas exposed to polluted air during acute HSK.

HSK is an immunopathological process involving increased immune mediators and inflammatory immune cell recruitment, including neutrophils and T cells, into the cornea, damaging the corneal tissue (38, 55). In concordance with these studies, we found an overwhelming corneal influx of leucocytes including neutrophils, inflammatory monocytes, dendritic cells, and T cells in HSV-1 infected mice exposed to air pollution with an increased HSK severity compared to non-exposed infected mice. These results suggest an increased corneal inflammatory response in correlation to a severe HSK, with low capacity to control viral load during early HSK in corneas exposed to air pollution.

Dendritic cells (DCs) interact with HSV-1 through toll-like receptors (TLRs) 4, 7, 8, and 9 to orchestrate an innate and adaptive anti-HSV-1 immune response (19). The increased number of corneal DCs and GR-1+ cells observed in mice exposed to polluted air during HSK could be caused by the increased viral load, contributing to an exacerbated pro-inflammatory response with the secretion of IFN-γ and TNF-α. Corneal DCs that migrate to dLNs initiate CD4+ effector T cells response during the acute phase of HSK (56). Thus, a higher number of resident corneal activated DCs may have direct implications on the T cell response development in local dLNs after HSV-1 infection.

Finally, we evaluated the changes in the adaptive immune response during acute HSK. Our first evident macroscopic sign was the significant increase in the submandibular dLNs area size in normal mice and at 7 and 14 days of HSK, suggesting an extended HSV-1 infection during HSK in mice exposed to polluted air. Then, we evaluate the CD4+ T cell effector response (T helper (Th) -1 and -17) and regulatory T cells (Tregs) by flow cytometry in local draining lymph nodes (dLNs) to determine the effects of chronic exposure to polluted air in the adaptive immune response during HSK. Our results showed a more immunogenic T cell response with a clear shift toward effector T cells response T (helper)h-1 over regulatory T cells (Tregs) at day 7 and both, Th1 and Th17 over Tregs in local dLNs at day 11 HSK, which correlates with worse outcomes in mice exposed to polluted air. Although we did not observe significant changes in the Th17 or Treg production in normal non-infected dLN, an increased dLN size and Th1 response was noted, indicating that this type of response prevailed during air pollution exposure. It was demonstrated that Th1 type response induces tissue damage in HSK promoted by the presence of corneal IFN-γ and TNF-α (57), while Th17 response also contributes to the pathogenesis of HSK following the first week after Th1 response (55), while Th17 response also contribute to the pathogenesis of HSK following first week after Th1 response (57). Moreover, PM from polluted air can promote effector T cells and aggravates autoimmune diseases, chronic obstructive pulmonary disease (COPD) or asthma, among others (43, 58–60). Thus, our data are aligned with these findings and supports the key role of effector T cell response (Th1 and Th17) in worsening the clinical outcomes on mice exposed to air pollution. Another important T cell contribution to the HSK severity is immune modulation made by the presence of Treg in the cornea during HSV-1 infection to control lesion severity by preventing inflammatory effector T cells, neutrophils and inflammatory monocytes trafficking to the site of infection (18); thus, the reduced corneal Tregs observed here at day 11 of HSK in polluted air exposed mice is potentially causing the increased immune cell infiltration and keratitis severity.

Together, these findings provide strong support for the notion that mice exposed to air pollution induce corneal DCs activation, exacerbating corneal inflammation by promoting a more immunogenic T cell type response in dLNs; however, the molecular and functional mechanisms involved will require further investigation.

In conclusion, the altered inflammatory profile induced by urban polluted air has profound long-term negative consequences on the host’s ability to respond to ocular pathogens and provides an understanding of the persistent and progressive nature of the immune response alterations during acute HSK. Our study underlines the potential impact of chronic exposure to urban air pollution from big cities in the clinical outcomes and immune responses to ocular inflammatory diseases.

## Data Availability Statement

The datasets presented in this study can be found in online repositories. The names of the repository/repositories and accession number(s) can be found in the article/**Supplementary Material**.

## Ethics Statement

The animal study was reviewed and approved by the Institutional Committee for the Care and Use of Laboratory Animals (CICUAL), University of Buenos Aires, in concordance to the Animal Care Guidelines from the National Institute of Health (USA) and the Association for Research in Vision and Ophthalmology (ARVO, USA) resolution on the use of animals in research.

## Author Contributions

VS is the first author of this work and the main contributor of this work. VS was in charge of the experimental design and performed the *in vivo* mouse models, sample management for further ELISA, immunofluorescence staining and flow cytometry assays, and also troubleshooting experimental issues, and data analysis and interpretations. VS also wrote the manuscript, made the figures and added the revisions suggested by the other authors. JT helped VS with the *in vivo* mouse models, sample management, and also the performing of the ELISA, immunofluorescence staining, and flow cytometry assays. JT also contributed to the manuscript writing and further revisions. GZ performed the clinical evaluations and ELISA measurements from corneal swabs. RI helped on the ELISA assay measurements and data analysis. EI contributed to the study by performing the PCR assay for viral DNA determination and data analysis. PC provided guidance and helped us in the discussion. AB was the PI in charge on conducting this research, contributing to the experimental designs, discussion, and troubleshooting any issues. AB also got the funding for the project, provided the workspace, reagents, equipment, supplies, and approved the protocols for chemicals handling, animal care, etc. AB made the final revisions of the manuscript. All authors contributed to the article and approved the submitted version.

## Funding

This study was supported by UBACyT founding #20020190100249BA (A.B.); Georg-Hannelore Zimmermann Foundation, München, Germany (A.B.), PICT 2017-1309, PICT 2017-4549 form National Agency for Promotion of Science and Technology, Argentina.

## Conflict of Interest

The authors declare that the research was conducted in the absence of any commercial or financial relationships that could be construed as a potential conflict of interest.
