# Supplementary figures and images for: Polluted Air Exposure Compromises Corneal Immunity and Exacerbates Inflammation in Acute Herpes Simplex Keratitis
Source: Front Immunol. 2021 Feb 25;12:618597. doi: 10.3389/fimmu.2021.618597 (PMC8025944; doi:10.3389/fimmu.2021.618597)

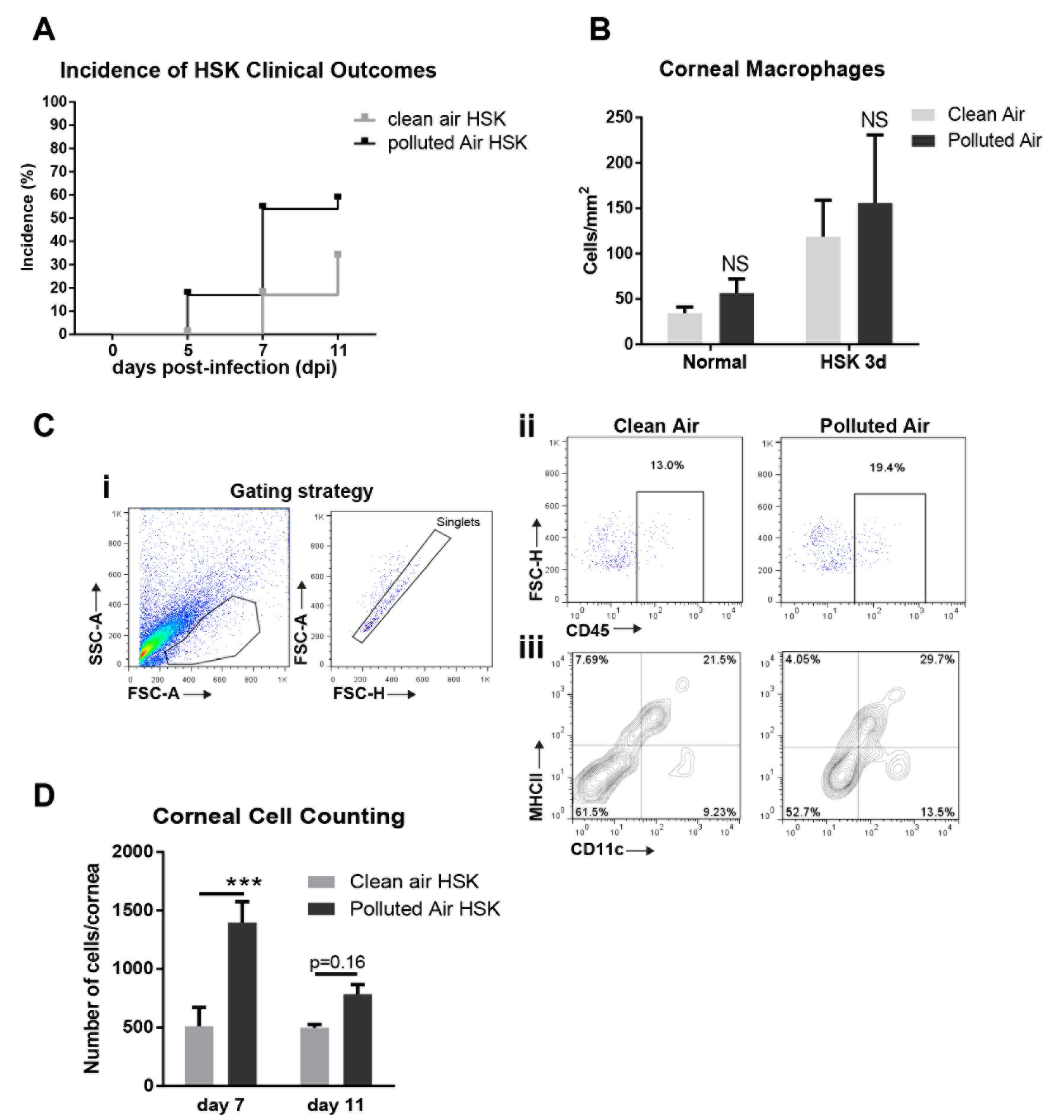

Supplement: Supplementary file 1 [file Image_1.tif]
