# Supplementary material for: Polluted Air Exposure Compromises Corneal Immunity and Exacerbates Inflammation in Acute Herpes Simplex Keratitis
Source: Front Immunol. 2021 Feb 25;12:618597. doi: 10.3389/fimmu.2021.618597 (PMC8025944; doi:10.3389/fimmu.2021.618597)
Supplement: Supplementary file 5 [file Table_1.docx]

Supplementary Material

**Supplementary Figure 1.** HSV-1 keratitis clinical outcomes incidence (**A**). Percentage of incidence showing clinical outcomes from group of polluted air-exposed mice (black line and square) and clean air-exposed mice (grey line and square) at day 0, 5, 7 and 11 post-infection (dpi). Total mice per group, N=24. B- Corneal Macrophages quantification (**B**). Macrophages (F4/80+) quantification by immunofluorescence staining in normal uninfected corneas and at day 3 HSK from mice exposed to clean air (grey bar) and polluted air (black bar). Graph results are presented as mean ± standard error of the mean (SEM). NS: statistically non-significant.

**Supplementary Figure 2.** Corneal draining lymph node immune cell subpopulations analysis by flow cytometry in uninfected mice exposed to clean or polluted air. T cell response Th1 (IFN-γ+) (**i**), Th17 (IL-17A+) (**ii**) and regulatory T cells, Treg (CD25+, FoxP3+) (**iii**) in local lymph nodes from normal mice exposed to clean air (grey bar) or polluted air (black bar) expressed as percentage of gated CD3+ CD4+ T cells. Graph results are presented as mean ± standard error of the mean (SEM). * p<0.05, differences between groups are statistically significant. NS: differences between groups are not statistically significant.

**Supplementary Figure 3.** Corneal draining lymph node immune cell subpopulations analysis by flow cytometry during acute HSV-1 keratitis on mice exposed to clean or polluted air. T cell response Th1 (IFN-γ+) (**i**), Th17 (IL-17A+) (**ii**) and regulatory T cells, Treg (CD25+, FoxP3+) (**iii**) in local lymph nodes from HSV-1 infected corneas at 7dpi from mice exposed to clean (grey bar) or polluted air (black bar) expressed as percentage of gated CD3+ CD4+ T cells. Graph results are presented as mean ± standard error of the mean (SEM). * p<0.05, **p<0.01, differences between groups are statistically significant. NS: differences between groups are not statistically significant.
